# Differential gene expression in brain tissues of aggressive and non-aggressive dogs

**DOI:** 10.1186/1746-6148-6-34

**Published:** 2010-06-16

**Authors:** Jørn Våge, Tina B Bønsdorff, Ellen Arnet, Aage Tverdal, Frode Lingaas

**Affiliations:** 1 Division of Genetics, Department of Basic Sciences and Aquatic Medicine, Norwegian School of Veterinary Science, Oslo, Norway; 2 Norwegian Institute of Public Health, Oslo, Norway

## Abstract

**Background:**

Canine behavioural problems, in particular aggression, are important reasons for euthanasia of otherwise healthy dogs. Aggressive behaviour in dogs also represents an animal welfare problem and a public threat. Elucidating the genetic background of adverse behaviour can provide valuable information to breeding programs and aid the development of drugs aimed at treating undesirable behaviour. With the intentions of identifying gene-specific expression in particular brain parts and comparing brains of aggressive and non-aggressive dogs, we studied amygdala, frontal cortex, hypothalamus and parietal cortex, as these tissues are reported to be involved in emotional reactions, including aggression. Based on quantitative real-time PCR (qRT-PCR) in 20 brains, obtained from 11 dogs euthanised because of aggressive behaviour and nine non-aggressive dogs, we studied expression of nine genes identified in an initial screening by subtraction hybridisation.

**Results:**

This study describes differential expression of the *UBE2V2 *and *ZNF227 *genes in brains of aggressive and non-aggressive dogs. It also reports differential expression for eight of the studied genes across four different brain tissues (amygdala, frontal cortex, hypothalamus, and parietal cortex). Sex differences in transcription levels were detected for five of the nine studied genes.

**Conclusions:**

The study showed significant differences in gene expression between brain compartments for most of the investigated genes. Increased expression of two genes was associated with the aggression phenotype. Although the *UBE2V2 *and *ZNF227 *genes have no known function in regulation of aggressive behaviour, this study contributes to preliminary data of differential gene expression in the canine brain and provides new information to be further explored.

## Background

The domestic dog (*Canis familiaris*), with its more than 400 recognised breeds [1], displays great variation in behaviour phenotypes. Favourable behaviour is important for well-being and negative traits such as aggression may ruin the owner-dog relationship and lead to relinquishment to shelters or even euthanasia of otherwise healthy dogs [2,3]. Behavioural traits result from an interaction of both genetic and environmental factors. Breed-specific behavioural traits such as hunting, herding and calmness/aggression are, however, evidence of a large genetic component and specific behaviours show high heritabilities [4-8].

Traditionally, rodents have provided the most important models for studies of genes involved in human mental illness. With the whole-genome sequencing of the modern dog completed [9], a new, efficient model for genetic studies has emerged. The dog suffers from a number of behavioural disorders and many of these have human parallels that are treated with the same medicines [10-14]. Canine patients from breeds with an increased risk of aggression, anxiety, and stereotypic behaviours provide important resources for genetic research.

Functional genome studies can be performed efficiently with cDNA microarrays [15] or subtraction hybridisation methods [16]. These are important methods to discover new associations between phenotype and expression of specific genes. However, understanding the nature of gene expression demands both the replication of initial results as well as further exploration of expression in additional samples and individuals. Detailed exploration of differential gene expression is most often performed with PCR (qRT-PCR) [17,18]. Besides generating important basic biological knowledge, canine genetic studies and a linking of specific genes to aggression may lead to the detection of markers to be used in breeding. Also such information will be important in the development of new drugs for treatment of behavioural disorders, both in animals and man.

Few studies have addressed gene expression in different canine brain parts. Saetre et al. [19] explored region-specific gene expression in canids and Lindberg et al. [20] described differential expression in different regions of the fox brain. In the present investigation we studied the expression of nine (Table 1) genes in different brain parts of aggressive and non-aggressive dogs by qRT-PCR. An initial PCR-based cDNA subtraction method, with the amygdala of an aggressive dog as the primary target, indicated that these genes were expressed differentially. The purpose of the work was to study gene-specific differences in expression between brain parts, and between aggressive and non-aggressive dogs.

**Table 1:** Genes and real-time amplification products

Gene symbol	Gene name	Accession no.	Gene function*	Primer	Amplicon size (bp)	PCR efficiency
**Target genes**						
*SCD*	stearoyl-CoA desaturase	XM_543968.2	synthesis of unsaturated fatty acids	Forward: CTCTACGGATATCGCCCTTACG	106	1.93
				Reverse: GAAAGGAGTGGTGGTAGTTGTGG		
					
*TMEM47*	transmembrane protein 47	NM_001003045.1	cellular component - plasma membrane	Forward: TGGTGGGTTTGATTTCTATCTGC	123	1.94
				Reverse: TGAACTTGATTGGGTAAAGGACC		
					
*ATP5A1*	ATP synthase	XM_861390.1	catalyzes ATP synthesis	Forward: TCCATGACGGAACTGATGAA	165	1.94
				Reverse: ACTGAAGTGGAGCAGCATCA		
					
*CALM 2*	calmodulin2	XM_859583.1	calcium ion binding	Forward: GATGAAATGATCAGGGAAGCA	178	1.93
				Reverse: AGCAGGGGGAAACCTTTTAC		
					
*UBE2V2*	TMEM189-UBE2V1	XM_544068.2	small conjugating protein ligase activity	Forward: AGGAGGAAAAATGCAGCACA	144	1.92
					
				Reverse: AACACACATGCTTCCACAGC		
					
*ZNF 227*	zinc finger protein 227	XM_850112.1	nucleic acid binding, zinc ion binding	Forward: CATCTTCCCTTCAAGCCAGA	119	2.1
				Reverse: TTTCGAAGTGTCTCCATCTCA		
					
*LOC488837*	similar to T05C3.2	XM_545955.2	apoptosis, protein-protein interactions**	Forward: CCGGAAAATTGCTAACATTGA	115	1.91
				Reverse: CAGCTTCTCTTGGGCTTCTG		
					
*RIMBP2*	RIMS binding protein 2	XM_543356.2	interaction in synaptic-vesicle release and presynaptic voltage-gated calcium channels***	Forward: CAGCTTCTGAGACAAGGCTTCC	124	1.97
				Reverse: TCGGGTCGTAATCATACAGGG		
					
*GLT1*	solute carrier family 1 member2	NM_001003138	synaptic clearance of glutamate	Forward: GGTCATCCTGGTCTTGGCTA	259	1.74
				Reverse: CTCCGTCACAGTCTCGTTCA		
					
**Endogenous control genes**						
*LOC482993 (RPL32)*	similar to 60S ribosomal protein L32	XM_540107	protein binding, structural constituent of ribosome	Forward: ATGCCCAACATTGGTTACGG Reverse: CTCTTTCCACGATGGCTTTG	181	1.84
					
*HNRNPH1*	heterogeneous nuclear ribonucleoprotein H1	XM_852029.1	RNA binding	Forward: CTCACTATGATCCACCACG Reverse: TAGCCTCCATAACCTCCAC	151	1.81

* Function by NCBI Entrez Gene or Gene Ontology for given gene or homologue

** Possible functions linked to conserved domains NACHT and WD40

*** Function by [43]

## Results

### Initial screening with cDNA subtraction assays

In the initial screening, the first of two parallel cDNA subtraction protocols identified 40 genes (Additional file 1) that were up-regulated in aggressive amygdala compared to non-aggressive amygdala. Six of these genes (*SCD*, *TMEM47*, *ATP5A1*, *CALM2*, *UBE2V2 *and *GLT1*) were tested with qRT-PCR in the brains of 20 dogs. The second parallel assay identified 46 genes up-regulated in the same aggressive amygdala versus frontal cortex, and five (*SCD*, *CALM2*, *ZNF227*, *LOC488837 *and *RIMBP2*) of these were tested with qRT-PCR. Two of the genes, *SCD *and *CALM2 *were common to both initial assays.

### qRT-PCR

Up-regulation detected by the initial screenings was tested by qRT-PCR in a broader database of unrelated dogs. Comparison of the aggressive and non-aggressive dogs in the extended samples of 20 dogs showed that there was a significant association of *UBE2V2 *(p = 0.0274, SEM = .3780, OR = 2.3) and *ZNF227 *(p = 0.0074, SEM = .4053, OR = 3.0) with the odds ratio (OR) for aggressive behaviour. Odds ratio expresses the relative change in odds that is associated with one unit change in gene expression. For the other seven genes no significant effect on the phenotype was detected. Eight of the nine investigated genes showed significant gene expression differences between the amygdala, frontal cortex, hypothalamus, and parietal cortex (Table 2). *LOC488837 *showed up-regulation (p = 0.0006) with qRT-PCR and thus supported the initial result from the amygdala versus frontal cortex assay. Five of the nine studied genes were up-regulated in male compared to female (Table 3).

**Table 2:** Tissue effect on expression

Gene	Effect of tissue on expression*	p-values** (SEM)
***SCD***	H > 1.28 > A	0.0757** (.1373)
	H > 1.32 > P	0.0449** (.1373)
		
***TMEM47***	A > 1.43 > F	0.0003 (.0956)
	A > 1.78 > P	< 0.0001 (.0956)
	F > 1.25 > P	0.0244** (.0956)
	H > 1.49 > F	0.0001 (.0982)
	H > 1.85 > P	< 0.0001 (.0982)
		
***ATP5A1***	F > 1.49 > A	0.0025 (.1278)
	F > 1.48 > H	0.0038 (.1314)
	P > 1.33 > A	0.0303** (.1278)
	P > 1.32 > H	0.0406** (.1314)
		
***CALM2***	A > 1.96 > H	< 0.0001 (.0874)
	F > 2.18 > H	< 0.0001 (.0874)
	P > 1.92 > H	< 0.0001 (.0874)
		
***UBE2V2***	F > 1.22 > H	0.0670** (.1072)
		
***LOC488837***	A > 1.43 > F	0.0006 (.0997)
	A > 2.16 > H	< 0.0001 (.1025)
	A > 1.60 > P	< 0.0001 (.0997)
	F > 1.51 > H	0.0001 (.1025)
	P > 1.32 > H	0.0043 (.1025)
		
***RIMBP2***	A > 2.08 > H	< 0.0001 (.1061)
	F > 1.98 > H	< 0.0001 (.1061)
	P > 1.75 > H	< 0.0001 (.1061)
		
***GLT1***	F > 2.34 > H	< 0.0001 (.0974)
	P > 2.06 > H	< 0.0001 (.0974)
	A > 2.37 > H	< 0.0001 (.0974)

* Fold effect of tissue on expression, A = amygdala, F = frontal cortex, H = hypothalamus, and P = parietal cortex, highest > relative expression > lowest.

** Not significant when applying Bonferroni correction (α = 0.05, n = 9 gives 0.05/9~0,006)

**Table 3:** Sex effect on expression

Gene	Effect of sex on expression*	p-values** (SEM)
***ATP5A1***	male > 1.20 > female	0.0569** (.0955)
		
***CALM2***	male > 1.23 > female	0.0014 (.0635)
		
***UBE2V2***	male > 1.36 > female	0.0002 (.0779)
		
***ZNF227***	male > 1.17 > female	0.0556** (.0802)
		
***GLT1***	male > 1.19 > female	0.0160** (.0708)

* Fold effect of sex on expression, highest > relative expression > lowest.

** Not significant when applying Bonferroni correction (α = 0.05, n = 9 gives 0.05/9~0,006)

## Discussion

The initial cDNA subtraction assays, from two dogs, revealed 83 differentially-expressed genes. Nine of these were tested with quantitative real-time PCR (qRT-PCR) in the samples from 20 dogs and revealed that *UBE2V2 *and *ZNF227 *expression was significantly associated with risk of aggression (though not significant with conservative Bonferroni correction p ~ 0.006). The up-regulation of *LOC488837 *in the amygdala versus frontal cortex supported the initial analysis. The relatively low number of initially-detected genes that showed up-regulation when tested with qRT-PCR may be a result of the target molecules being rare transcripts with low degrees of differential expression. Phenotypic differences in the dogs of the two data sets, sampling and the heterogeneous nature of the brain tissues may also have contributed to the different results from the two methods. Consequently, in our study, the initial subtraction assays worked as small-scale screenings for potential transcripts. The real-time study included more individuals than the initial assays and would as such have had much stronger statistical validity.

Quantitative real-time PCR is a sensitive method for detection of genes showing low levels of expression differences. For most of the genes in the study the estimated expression differences between phenotype, sex and brain parts were relatively small ( < 2X). Wurmbach et al. [21] have shown that most regulated genes in brain tissues in mouse fall within this range. The heterogeneous nature of brain regions with a large variety of cell populations that are closely intermingled may result in only small fold-changes for biologically significant expression differences.

The analysis shows that up-regulation of *UBE2V2 *(p = 0.0274) and *ZNF227 *(p = 0.0074) are significantly associated with increased OR for aggressive behaviour. Zinc finger protein 227 (nucleotide blast, NCBI) is a gene in the Krüppel-type zinc finger family. In man, this is a large gene family with Krüppel-associated box (KRAB) motifs and the KRAB-ZNF proteins are predicted to function as transcriptional regulators [22]. Functionally, the *UBE2V2 *gene has been proposed to participate in cell proliferation, protein polyubiquitination, regulation of DNA repair, regulation of progression through cell cycle, protein modification, and the ubiquitin cycle. Disposition for aggressive behaviour is most probably polygenic and influenced by several different neurotransmitter and hormonal systems. Our results indicate that *UBE2V2 *is involved in pathways associated with aggressive behaviour and that *ZNF227 *is involved in transcription regulation, possibly associated with behavioural change.

Analyses of different brain tissues yield, as expected, differential gene expression between the tissues. Although these differences have no established link to any behavioural phenotypes they are a contribution to the limited reports of canine brain gene expression. The studied genes are to a great extent down-regulated in hypothalamus, with the exception of *SCD *and *TMEM47 *(Table 2). Interestingly, SCD was one of the genes that were identified in both our initial independent studies. Saetre et al. [23] also showed down-regulation of a number of hypothalamic genes, compared to amygdala and frontal lobe, while *SCD *was reported by Dridi et al. [24] to be highly expressed in chicken hypothalamus. *SCD *is an iron-containing enzyme that catalyses a rate-limiting step in the synthesis of unsaturated fatty acids and Yamasaki et al. [25] recently showed stearoyl-CoA desaturase 4 to be up-regulated in brain tissues from schizophrenic patients. Another observation of interest is the large number of genes showing expression differences between brain tissues. This observation is supported by Saetre et al. [26] who found that brain tissue differences exceeded the difference between different canid species, and by Strand et al. [27] who showed that gene expression in equivalent brain regions of mice and humans is more alike than different regions within the same species. Kennerly et al. [28] revealed regional expression differences in the brain but did not find marked differences among breeds or individual dogs.

*CALM2 *(Calmodulin2) functions include roles in growth and the cell cycle as well as in signal transduction and the synthesis and release of neurotransmitters. In our study, *CALM2 *showed lower expression in hypothalamus compared to the three other tissues (Table 2), which coincides with the findings of Saetre et al. [29] for *CALM1*. CALM2 also showed differential expression in both our initial independent studies.

*GLT1*, the gene for which the largest differences in expression between tissues were observed (A > 2.37 > H), is the main gene responsible for clearance of synaptic glutamate, which is the major excitatory neurotransmitter in the central nervous system. Altered expression of *GLT1 *and different splice-variants have been linked to a number of neuropsychiatric disorders, including schizophrenia and mood and anxiety disorders [30].

*LOC488837*, is so far annotated as a model protein. The canine transcript has only been reported in a brain cDNA library (NCBI, Unigene). Additionally, in man the transcript of the ortholog sequence *KIAA1239 *has mainly been identified in brain tissues (NCBI, Unigene). Our analysis shows that the gene is up-regulated in the amygdala compared to the three other tissues and in the frontal- and parietal cortex compared to hypothalamus. (Table 2).

*RIMBP2 *(RIMS binding protein 2) shows a 1.75 - 2.08-fold up-regulation in amygdala, frontal and parietal cortex compared to hypothalamus. These results are supported by a study of rats [31] showing that *RIMBP2 *is highly expressed in telencephalon compared to other brain regions.

In our analysis *UBE2V2 *was expressed at a low level in the hypothalamus compared to frontal and parietal cortex. *ATP5A1 *also showed differentiated expression with down-regulation in amygdala and hypothalamus compared to both of the cortex areas.

Our study identified gender differences in expression of *ATP5A1*, *CALM2*, *GLT1*, *UBE2V2*, and *ZNF227 *(Table 3). We are not aware of any other reports of gender-related differences in gene expression between canine brain parts. The genes *ZNF227 *and *UBE2V2 *show a significant up-regulation in both the aggressive group and the male group of dogs. The fact that both male and female dogs were equally represented in the aggressive and non-aggressive groups, and the use of a statistical model accounting for both effects, support the results.

In the study we have presented raw significance levels as well as the Bonferroni values corrected for multiple testing. Each p-value is presented for the reader to have full insight in the results. It could, however, be discussed if the Bonferroni correction in this case is a too conservative correction. We consider the initial analysis as a method to establish hypotheses about differential expression in a set of samples. The testing of the initial results with a second method in an extended population of unrelated dogs should give a good basis for assessing the initial results.

Phenotyping of the included dogs was based on questionnaires [32]. Canine aggression is a complex phenotypic trait, which probably has a complex genetic regulation and environmental influence. The aggressive dogs in this study were grouped on the basis of shared aggressive behaviours toward humans.

## Conclusions

This study identifies the *UBE2V2 *and *ZNF227 *expression as being associated with aggression. Since studies reporting differential gene expression in canine brain tissue are few in numbers, we have included results elucidating the expression of eight genes in canine brain, including a novel finding of gene expression of *LOC488837*. Of the same reason we also report findings of differential expression between male and female dogs. We are not aware of any reports based on this high number of brain samples (n = 20). The statistical power of 20 samples is, however, not optimal and call for some caution when interpreting the result. Our work contributes with preliminary data in an important area of research that can improve the understanding of functional aspects of aggression.

## Methods

### Samples

Brain samples from 11 dogs *(Canis familiaris) *euthanised for aggressive behaviour and nine brains from non-aggressive dogs were sampled in collaboration with veterinary clinics. All dogs were euthanised (anesthetised with i.m. medetomidine+ketamine and cardiac arrest induced with pentobarbital) in accordance with the owner, and for reasons other than participation in the study. For each dog included in the study, a questionnaire [32] answered by the dog's owner, describing the dog's behavioural traits, accompanied the samples. The questionnaire also included information of disease status and indicated that all dogs included were healthy. The aggressive dogs, 5 English cocker spaniels (3 male, 2 female), 1 German shepherd dog (female, "tester" in initial subtractive hybridisation assay), 1 mixed breed (male), 1 flat-coated retriever (male), 1 English setter (male), 1 Dutch shepherd dog (male) and 1 dachshund (female), all had severe behavioural problems, characterised by frequent growling, snapping and biting, directed towards both family members and strangers. The non-aggressive dogs, 3 English cocker spaniels (2 male, 1 female), 2 German shepherd dogs (1 male, 1 female ("driver" in initial subtractive hybridisation assay)), 1 mixed breed (male), 1 Norwegian lundehund (female), 1 small Munsterlander (male) and 1 tervueren (male), were reported to show excellent behaviour. The age of the included dogs, mean = 49 months (min. 15, max. 102) for aggressive, and mean = 77 months (min. 24, max.158) for non-aggressive.

Tissue samples from 4 brain compartments reported to be involved in emotional response, including aggression [33-37], frontal cortex, parietal cortex, hypothalamus and amygdala were harvested < 1 hr post-mortem and immediately frozen in liquid nitrogen or immersed into RNA-later™(Ambion). Samples were stored at -80°C or -20°C respectively.

### RNA isolation

Total RNA used in the cDNA subtraction assays was isolated by acid guanidinum thiocyanate-phenol-chloroform extraction [38]. In addition mRNA was extracted using the Dynabeads^® ^mRNA Purification Kit (Dynal^® ^Biotech). Isolation of total RNA used in quantitative real-time PCR (qRT-PCR) analysis was performed with a modified TRIzol^® ^protocol (Invitrogen™). In brief, 30mg tissue was homogenised in 500ml TRIzol^® ^reagent (Invitrogen™) using a Retsch MM300 mixer mill (Retsch GmbH, Germany) and Tungsten Carbide Beads (Qiagen). Samples were further centrifuged, the clear homogenate was separated and incubated, 200 μl chloroform were added, and the mixture shaken and incubated. Phases were separated and to the RNA phase were added 500 μl isopropanol, and the mixture again incubated. Finally, samples were centrifuged and pellets washed with ethanol, left to partly air dry, and redissolved in 30 μl of RNase-free water at 60°C. RNA was stored at -80°C. All quantification of total RNA was performed with a NanoDrop™1000 Spectrophotometer (Thermo Fisher Scientific Inc.).

### Target gene selection

An initial screen for differentially-expressed gene products was performed with a Clontech PCR-Select™ cDNA Subtraction Kit (BD Biosciences Clontech) using two different sample sets. One identified transcripts differentially expressed (up-regulation) in the amygdala of an aggressive German shepherd bitch (tester) versus amygdala of a non-aggressive German shepherd bitch (driver). In a parallel assay, transcripts were identified that were differentially expressed (up-regulation) in the amygdala of the same aggressive dog (tester) compared to frontal cortex (driver), where the frontal cortex was a pooled sample of both the aggressive and non-aggressive dogs. The standard protocol from Clonetech, with approximately 2 μg mRNA input, was used. The cDNA subtraction assay is basically not a quantitative method, but is aimed at detecting differentially-expressed transcripts between two RNA populations. The method is described to identify also rare transcripts, with a normalisation step (equalising the numbers of abundant and rare transcripts due to hybridisation kinetics) and a relative enrichment of differentially-expressed target transcripts during subtraction [39,40]. Following subtraction, a selective PCR step amplifies target molecules, thus increasing the chance of detecting transcripts for further investigation.

Resulting PCR products from the subtracted cDNAs were cloned by TOPO TA cloning^® ^(Invitrogen™ ). Selected clones were prepared with Templiphi (Invitrogen™) and sequenced by the MegaBACE™ 1000 DNA Analysis System (Amersham Biosciences) using the DYEnamic™ ET Dye Terminator Kit (Amersham Biosciences). Obtained sequences were identified by BLAST search (web search at "nucleotide blast", NCBI) and a total of 83 genes (the three genes *SCD*, *CALM2 *and *COX1 *were common to both assays) were identified as up-regulated in the cDNA subtraction assays (Additional file 1, the number of sequenced clones from the amygdala/amygdala assay was 65 (40 unique and 6 mitochondrion and from the amygdala/frontal assay 81 clones were obtained (46 unique and 9 mitochondrion)). From these, 9 genes were selected for verification of the initially-detected up-regulation and for further investigation. *SCD *and *CALM2 *were gene products detected commonly in both initial assays. *GLT1 *(together with *CALM2*) is a gene involved in the glutamate signalling pathway. *TMEM47*, *ATP5A1*, *UBE2V2*, *ZNF227*, *LOC488837 *and *RIMBP2 *were randomly chosen to verify the initial assays. The expression of the nine target genes was further explored by qRT-PCR analysis (Table 1) across four brain regions - amygdala, frontal cortex, hypothalamus and parietal cortex - of 20 dogs (including the two German shepherd dogs analysed in the initial subtractive hybridisation assays).

### Quantitative real-time PCR

qRT-PCR was carried out in an Applied Biosystems 7900HT Fast Real-Time PCR system (Applied Biosystems, USA) using a Platinum^® ^SYBR^®^Green qPCR SuperMix UDG with ROX (Invitrogen™). This was a two-step qRT-PCR procedure that used Superscript™ III First-Strand Synthesis SuperMix for qRT-PCR (Invitrogen™) to generate cDNA from a sample of 11ng total RNA. The experiment was carried out according to the manufacturer's protocols with adjustments for smaller volumes; end volume of the real-time reaction was 20 μl. To avoid pipetting small volumes the cDNA were diluted to allow input of 8 μl template, corresponding to 0.35ng cDNA, in the real-time reaction. All samples were analysed in duplicate from the RT reactions (cDNA synthesis) and throughout the experiment, along with water and negative controls lacking RT enzyme. For individual genes, all samples were run on the same 96-well plate. Dissociation curve analysis was performed to rule out amplification of non-specific products. Temperature profiles for RT reactions: 25°C (10 min), 42°C (50 min), 85°C (5 min), chill on ice, 37°C (20 min, added *E. coli *RNase H), and real-time reactions: 50°C (2 min), 95°C (2 min), followed by 40 cycles of 95°C (15 sec), 60°C (1 min), and finally melting point analysis. The data were analysed with SDS 2.3 and post-analysed using RQ Manager 1.2 (Applied Biosystems), and Excel (Microsoft).

Real-time PCR amplification efficiency was examined by generating standard curves from serial dilutions of pooled cDNA from all four tissues with both case and control samples represented. A 125-fold range dilution with the most concentrated sample corresponding to cDNA derived from 0.88ng total RNA. Curves were generated by plotting the log _10 _of the quantity of each template dilution against the C _T _value obtained during amplification of each dilution. The dilution curves were linear for all gene products with R ^2 ^values in linear regression > 0.980. The estimated relative gene expression (RE) levels were calculated as 2 ^CT(endogenous) " CT (target)^, a variation of the Livak method [41], where we corrected for variation in amplification efficiency (E = 10 ^-1/slope^, Table 1.) using:

Thus we obtained an estimated relative gene expression normalised against an endogenous control gene (EC). Further, the values for the two different EC were normalised (in order to average numeric different values between different EC) with Z = X- μ/σ, before they were averaged. The same procedure was carried out for the second replicate of samples, and finally values for replicate one and two were also averaged. Thus for statistical analysis (SAS 9.1 BASE, SAS-PC System^® ^Version 9.1 for Windows, SAS Institute Inc., Cary, NC, USA, 2002) we had 80 averaged values representing 20 dogs x 4 different tissues. For analysis of expression differences between different brain tissues, and between sex the calculated values were natural log (ln) transformed before analysis, using a multiple regression model:

where u is the overall mean, a, b, c is the effect of each of the three tissues T2, T3, T4 relative to tissue T1 (reference), d is the effect of sex (S), f is the effect of phenotype (P) and e is the random error.

For analysis of risk of aggressive phenotype in relation to specific gene expression we used a logistic regression model:

where logit(P) is the logit of the phenotype-value ln(P/1-P)(aggressive/non-aggressive), u is the overall mean, a, b, c is the effect of each of the three tissues T2, T3, T4 relative to tissue T1 (reference), d is the effect of gene specific expression (E), and e is the random error.

### Differential expression

In the regression model all tissues and sex were coded 1/0 and the relative expressions could therefore be calculated from differential expression = exp ^coefficient^, where the coefficients (parameter estimate) for different tissues and sex is derived from the regression analysis. The logit model gives the OR for aggressive behaviour compared to non-aggressive.

### Endogenous control genes

Initially 8 different genes (*ACTB*, *HNRNPH1*, *HPRT1*, *RPL32*, *RPS5*, *TBP*, *TFRC*, and *TKT*) were tested as potential endogenous controls. Selection of genes used in the analysis was facilitated by the software geNorm [42], with adjustments for PCR amplification efficiency (E) when generating input data (E ^-ΔCt^), identifying *RPL32 *and *HNRNPH1 *(M = 0.40 and V _2/3 _= 0.157) as sufficient genes to correct for loading differences of cDNA.

## Author's contributions

JV carried out molecular genetic studies and drafted the manuscript. TBB carried molecular studies and helped to draft the manuscript. EA carried out real-time PCR analysis. AT participated in the design of the study, performed statistical analysis and revised the manuscript critically for important intellectual content. FL conceived of the study, and participated in its design, statistical analysis and coordination and helped to draft the manuscript. All authors read and approved the final manuscript.

## Supplementary Material

Additional file 1 **Gene list of initially detected transcripts**. Gene list of initially detected transcripts. Gene IDs are putative human homologes (identified by Homologene, NCBI) of the identified canine sequences. Click here for file
