# Supplementary material for: Differential gene expression in brain tissues of aggressive and non-aggressive dogs
Source: BMC Vet Res. 2010 Jun 16;6:34. doi: 10.1186/1746-6148-6-34 (PMC2898780; doi:10.1186/1746-6148-6-34)
Supplement: Additional file 1 — Gene list of initially detected transcripts. Gene list of initially detected transcripts. Gene IDs are putative human homologes (identified by Homologene, NCBI) of the identified canine sequences. [file 1746-6148-6-34-S1.DOC]

Additional file 1

Gene list of initially detected transcripts, generated with IPA, Ingenuity Systems, Inc. Gene IDs are putative human homologes (identified by Homologene, NCBI) of the identified canine sequences.

| **List of genes detected up-regulated in amygdala versus amygdala cDNA subtraction assays** | | | | |
| --- | --- | --- | --- | --- |
| **Human ID** | **Symbol** | **Entrez Gene Name** | **Location** | **Type** |
| 4512 | COX1 | cytochrome c oxidase subunit I | Cytoplasm | enzyme |
| 4514 | COX3 | cytochrome c oxidase III | Cytoplasm | enzyme |
| 4519 | CYTB | cytochrome b | Cytoplasm | enzyme |
| 64794 | DDX31 | DEAD (Asp-Glu-Ala-Asp) box polypeptide 31 | Nucleus | enzyme |
| 1759 | DNM1 | dynamin 1 | Cytoplasm | enzyme |
| 4190 | MDH1 | malate dehydrogenase 1, NAD (soluble) | Cytoplasm | enzyme |
| 4860 | NP | nucleoside phosphorylase | Nucleus | enzyme |
| 5048 | PAFAH1B1 | platelet-activating factor acetylhydrolase, isoform Ib, alpha subunit 45kDa | Cytoplasm | enzyme |
| 5862 | RAB2A | RAB2A, member RAS oncogene family | Cytoplasm | enzyme |
| 6319 | SCD | stearoyl-CoA desaturase (delta-9-desaturase) | Cytoplasm | enzyme |
| 55585 | UBE2Q1 | ubiquitin-conjugating enzyme E2Q family member 1 | Unknown | enzyme |
| 7417 | VDAC2 | voltage-dependent anion channel 2 | Cytoplasm | ion channel |
| 26289 | AK5 | adenylate kinase 5 | Cytoplasm | kinase |
| 10552 | ARPC1A | actin related protein 2/3 complex, subunit 1A, 41kDa | Cytoplasm | other |
| 57035 | C1ORF63 | chromosome 1 open reading frame 63 | Unknown | other |
| 805 | CALM2 | calmodulin 2 (phosphorylase kinase, delta) | Plasma Membrane | other |
| 977 | CD151 | CD151 molecule (Raph blood group) | Plasma Membrane | other |
| 81631 | MAP1LC3B | microtubule-associated protein 1 light chain 3 beta | Cytoplasm | other |
| 4684 | NCAM1 | neural cell adhesion molecule 1 | Plasma Membrane | other |
| 27020 | NPTN | neuroplastin | Plasma Membrane | other |
| 5037 | PEBP1 | phosphatidylethanolamine binding protein 1 | Cytoplasm | other |
| 22902 | RUFY3 | RUN and FYVE domain containing 3 | Unknown | other |
| 6281 | S100A10 | S100 calcium binding protein A10 | Cytoplasm | other |
| 6744 | SSFA2 | sperm specific antigen 2 | Plasma Membrane | other |
| 6612 | SUMO3 | SMT3 suppressor of mif two 3 homolog 3 (S. cerevisiae) | Nucleus | other |
| 387522 | TMEM189-UBE2V1 | TMEM189-UBE2V1 readthrough transcript | Unknown | other |
| 59353 | TMEM35 | transmembrane protein 35 | Unknown | other |
| 83604 | TMEM47 | transmembrane protein 47 | Plasma Membrane | other |
| 54704 | PPM2C | protein phosphatase 2C, magnesium-dependent, catalytic subunit | Cytoplasm | phosphatase |
| 5515 | PPP2CA | protein phosphatase 2 (formerly 2A), catalytic subunit, alpha isoform | Cytoplasm | phosphatase |
| 54467 | ANKIB1 | ankyrin repeat and IBR domain containing 1 | Nucleus | transcription regulator |
| 57498 | KIDINS220 | kinase D-interacting substrate of 220 kDa | Nucleus | transcription regulator |
| 8672 | EIF4G3 | eukaryotic translation initiation factor 4 gamma, 3 | Cytoplasm | translation regulator |
| 1983 | EIF5 | eukaryotic translation initiation factor 5 | Cytoplasm | translation regulator |
| 498 | ATP5A1 | ATP synthase, H+ transporting, mitochondrial F1 complex, alpha subunit 1 | Cytoplasm | transporter |
| 57515 | SERINC1 | serine incorporator 1 | Plasma Membrane | transporter |
| 6506 | SLC1A2 | solute carrier family 1 (glial high affinity glutamate transporter), member 2 | Plasma Membrane | transporter |
| 29887 | SNX10 | sorting nexin 10 | Cytoplasm | transporter |
| 26519 | TIMM10 | translocase of inner mitochondrial membrane 10 homolog (yeast) | Cytoplasm | transporter |
| 9804 | TOMM20 | translocase of outer mitochondrial membrane 20 homolog (yeast) | Cytoplasm | transporter |
|  |  |  |  |  |
| **List of genes detected up-regulated in amygdala versus frontal cortex cDNA subtraction assays** | | | | |
| **Human ID** | **Symbol** | **Entrez Gene Name** | **Location** | **Type** |
| 51582 | AZIN1 | antizyme inhibitor 1 | Cytoplasm | enzyme |
| 4512 | COX1 | cytochrome c oxidase subunit I | Cytoplasm | enzyme |
| 51635 | DHRS7 | dehydrogenase/reductase (SDR family) member 7 | Unknown | enzyme |
| 60481 | ELOVL5 | ELOVL family member 5, elongation of long chain fatty acids | Unknown | enzyme |
| 2495 | FTH1 | ferritin, heavy polypeptide 1 | Cytoplasm | enzyme |
| 2820 | GPD2 | glycerol-3-phosphate dehydrogenase 2 (mitochondrial) | Cytoplasm | enzyme |
| 2992 | GYG1 | glycogenin 1 | Cytoplasm | enzyme |
| 4649 | MYO9A | myosin IXA | Cytoplasm | enzyme |
| 4538 | ND4 | NADH dehydrogenase, subunit 4 (complex I) | Cytoplasm | enzyme |
| 6319 | SCD | stearoyl-CoA desaturase (delta-9-desaturase) | Cytoplasm | enzyme |
| 5218 | PFTK1 | PFTAIRE protein kinase 1 | Nucleus | kinase |
| 5756 | TWF1 | twinfilin, actin-binding protein, homolog 1 (Drosophila) | Cytoplasm | kinase |
| 91452 | ACBD5 | acyl-Coenzyme A binding domain containing 5 | Unknown | other |
| 805 | CALM2 | calmodulin 2 (phosphorylase kinase, delta) | Plasma Membrane | other |
| 966 | CD59 | CD59 molecule, complement regulatory protein | Plasma Membrane | other |
| 2017 | CTTN | cortactin | Plasma Membrane | other |
| 3190 | HNRNPK | heterogeneous nuclear ribonucleoprotein K | Nucleus | other |
| 57495 | KIAA1239 | KIAA1239 | Unknown | other |
| 51088 | KLHL5 | kelch-like 5 (Drosophila) | Unknown | other |
| 3964 | LGALS8 | lectin, galactoside-binding, soluble, 8 | Extracellular Space | other |
| 116135 | LRRC3B | leucine rich repeat containing 3B | Unknown | other |
| 10150 | MBNL2 | muscleblind-like 2 (Drosophila) | Unknown | other |
| 10787 | NCKAP1 | NCK-associated protein 1 | Plasma Membrane | other |
| 257194 | NEGR1 | neuronal growth regulator 1 | Extracellular Space | other |
| 4897 | NRCAM | neuronal cell adhesion molecule | Plasma Membrane | other |
| 23504 | RIMBP2 | RIMS binding protein 2 | Plasma Membrane | other |
| 6711 | SPTBN1 | spectrin, beta, non-erythrocytic 1 | Plasma Membrane | other |
| 7102 | TSPAN7 | tetraspanin 7 | Plasma Membrane | other |
| 11091 | WDR5 | WD repeat domain 5 | Nucleus | other |
| 1636 | ACE | angiotensin I converting enzyme (peptidyl-dipeptidase A) 1 | Plasma Membrane | peptidase |
| 1363 | CPE | carboxypeptidase E | Plasma Membrane | peptidase |
| 5687 | PSMA6 | proteasome (prosome, macropain) subunit, alpha type, 6 | Cytoplasm | peptidase |
| 5803 | PTPRZ1 | protein tyrosine phosphatase, receptor-type, Z polypeptide 1 | Plasma Membrane | phosphatase |
| 22908 | SACM1L | SAC1 suppressor of actin mutations 1-like (yeast) | Cytoplasm | phosphatase |
| 1499 | CTNNB1 | catenin (cadherin-associated protein), beta 1, 88kDa | Nucleus | transcription regulator |
| 2023 | ENO1 | enolase 1, (alpha) | Cytoplasm | transcription regulator |
| 2971 | GTF3A | general transcription factor IIIA | Nucleus | transcription regulator |
| 221895 | JAZF1 | JAZF zinc finger 1 | Nucleus | transcription regulator |
| 57498 | KIDINS220 | kinase D-interacting substrate of 220 kDa | Nucleus | transcription regulator |
| 8031 | NCOA4 | nuclear receptor coactivator 4 | Nucleus | transcription regulator |
| 64324 | NSD1 | nuclear receptor binding SET domain protein 1 | Nucleus | transcription regulator |
| 7770 | ZNF227 | zinc finger protein 227 | Nucleus | transcription regulator |
| 162 | AP1B1 | adaptor-related protein complex 1, beta 1 subunit | Cytoplasm | transporter |
| 1314 | COPA | coatomer protein complex, subunit alpha | Cytoplasm | transporter |
| 84263 | HSDL2 | hydroxysteroid dehydrogenase like 2 | Cytoplasm | transporter |
| 6507 | SLC1A3 | solute carrier family 1 (glial high affinity glutamate transporter), member 3 | Plasma Membrane | transporter |
